# Primary Intestinal NK/T-cell Lymphoma Masquerading as Crohn’s Disease: A Report of Two Cases

**DOI:** 10.7759/cureus.77056

**Published:** 2025-01-07

**Authors:** Priya Jayakumar, Lavleen Singh, Vineet Ahuja, Rajni Yadav, Saumyaranjan Mallick

**Affiliations:** 1 Pathology, All India Institute of Medical Sciences, New Delhi, New Delhi, IND; 2 Gastroenterology, All India Institute of Medical Sciences, New Delhi, New Delhi, IND

**Keywords:** conundrum, crohn’s disease, extranodal, nk/t cell lymphoma, prognosis

## Abstract

Extranodal NK/T-cell lymphoma (ENKTL) most commonly affects the upper aerodigestive tract. Primary intestinal NK/T-cell lymphoma is extremely rare and poses a formidable challenge for both the gastroenterologists and pathologists. We herein report two cases of ENKTL. On histopathological and immunohistochemical examination of the resected specimens, a final diagnosis of primary intestinal NK/T-cell lymphoma was made. Due to significant overlap in clinical, endoscopic, and histopathological findings, this entity is often misdiagnosed as Crohn’s disease. Multiple endoscopic biopsies with the use of an elaborate immunohistochemical panel can help in diagnosis at an early stage, thereby improving the management and prognosis.

## Introduction

Extranodal NK/T-cell lymphoma (ENKTL) is of NK or T-cell lineage with vascular damage and destruction, prominent necrosis, a cytotoxic phenotype, and association with Epstein Barr virus (EBV) [1]. It has an extranodal presentation and most commonly affects the upper aerodigestive tract (nasal cavity, nasopharynx, paranasal sinuses, and palate) [2]. NK/T-cell lymphoproliferative disorders occurring in the gastrointestinal (GI) tract are extremely rare. They comprise 5.2% to 14.7% of all primary intestinal lymphomas [3]. Owing to its rarity and vague clinical symptoms, it poses a formidable challenge for both the gastroenterologists and pathologists. These cases are often clinically misdiagnosed as inflammatory bowel disease, and the inadvertent use of steroids may further complicate the clinical course and pathological diagnosis.

We report two cases of different age groups diagnosed as Crohn’s disease based on the clinical and endoscopic findings. A careful histopathological and immunohistochemical evaluation revealed extranodal NK/T-cell lymphoma of the colon. This is being discussed here to highlight the clinical, histopathological, and immunohistochemical features and diagnostic pitfalls with a detailed review of literature for this rare entity.

## Case presentation

Case 1

A 54-year-old male presented with a two-year history of large-bowel diarrhea with blood-mixed stools without urgency or tenesmus, along with dull, non-colicky, peri-umbilical abdominal pain and significant weight loss (30% actual weight). He also complained of prolonged episodes of fever between 100-102 degrees Fahrenheit (F) for one month that paralleled an increase in diarrheal stool frequency. Also, he had large joint symmetric polyarthritis for two years.

Colonoscopic examination revealed multiple scattered deep serpiginous ulcers from the descending colon to the cecum. Computed tomography (CT) enterography confirmed bowel wall thickening and vasa recta prominence of the transverse colon, hepatic flexure, and ascending colon without any mass formation. An early diagnosis of inflammatory bowel disease was rendered, and the patient received two courses of steroids with significant improvement in diarrhea and fever. His episodes of fever appeared to be disproportionate to that expected in inflammatory bowel disease, and there was no evidence of significant inflammation on histopathological examination of subsequent colonoscopic biopsy. Subsequently, he was admitted for evaluation of pyrexia of unknown origin with persistent daily fever (102°F) and bloody diarrhea. GeneXpert performed from colonoscopic biopsies ruled out evidence of active tuberculosis. Viral serology for HIV and syphilis was negative. There were no cutaneous markers of disseminated histoplasmosis. The patient developed leukopenia that worsened during the hospital stay. He was treated with empirical intravenous antibiotics and corticosteroids (hydrocortisone). A PET-CT was performed electively, which revealed pneumoperitoneum and multifocal thickening of the colon. We were, however, unable to retrieve the radiological images of the patient. The patient developed pain in the abdomen while admitted, and an abdominal X-ray performed showed perforation. He was subsequently taken up for emergency explorative laparotomy, and a subtotal colectomy was performed. 

**Figure 1:**
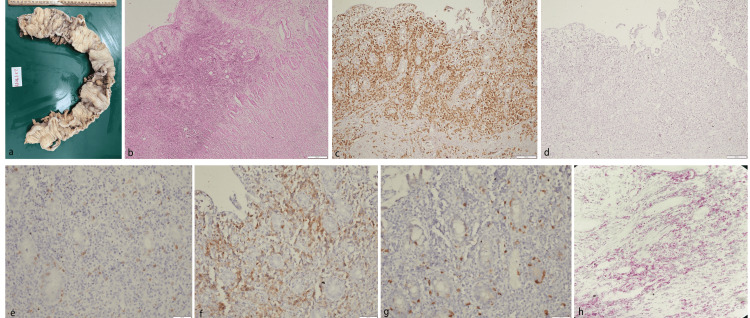
Case Report 1 a: gross specimen of intestine showing two diffuse ulceroinfiltrative thickened lesions with intervening normal-appearing mucosa (rest of the mucosa shows superficial ulcers); b: ulcerative lesion infiltrated by medium sized lymphoid cells up to muscularis propria (HE, x100); c: diffuse immunopositivity for CD2 (x200); d: negative for CD20 (x200); e: abberant loss of CD4 (x200); f: immunopositivity for in-situ hybridisation of Eber (x200); g: aberrant loss of CD8 (x200); and h: immunopositivity for mRNA scopy of Eber (x200)

Gross examination of the subtotal colectomy specimen showed multiple superficial and deep ulcers involving the ileum, cecum, ascending, and transverse colon. The base of the ulcers was filled with necrotic material and covered with slough. The bowel wall was firm and thickened. An ulcerated and thickened area was identified at the hepatic flexure measuring 1 x 0.8 cm. Another similar-looking area was identified at a distance of 4 cm distal to the first. In between these two lesions, intervening mucosa appeared unremarkable (skip lesion) (Figure 1).

Microscopic examination showed transmural infiltration by small- to medium-sized atypical lymphoid cells. There were superficial mucosal ulcerations as well as deep fissuring ulcerations extending up to the muscularis propria. The lesion had an angiocentric and angiodestructive pattern inducing extensive coagulative necrosis and mucosal ischemic changes. The atypical cells had coarse granular nuclear chromatin, inconspicuous nucleoli, and scant cytoplasm. In addition, twelve lymph nodes dissected from the attached fat were free of disease and showed reactive change only.

On immunohistochemical examination, the atypical cells showed diffuse positivity for T-cell markers CD3 and CD7 with focal immunopositivity for EBV. There was aberrant loss of CD4 and CD8. These cells were immunonegative for CD56, CD5, CD30, and CD20 and showed expression for TIA-1, perforin, and granzyme B. In situ hybridization studies were positive for EBV (Eber 1 and Eber 2). mRNA sequencing was also positive for amplified RNA sequencing of EBV (Figure 1). Extensive systemic and radiological examination did not reveal any nasal lesion, thereby excluding the possibility of a primary nasal origin. 

A final diagnosis of primary extranodal NK/T-cell lymphoma of the gastrointestinal tract was established. The patient, however, expired on the tenth day of admission, and further options of treatment could not be implored.

Case 2

An 18-year-old boy presented with chronic bloody diarrhea, urgency, tenesmus, periumbilical pain, and significant weight loss over the course of a year. Despite prior treatments, including antibiotics, the symptoms persisted. Colonoscopy showed multiple deep ulcers with patchy loss of vascular pattern and intervening skip areas from rectum to cecum. Histopathology of the biopsied ulcer showed features of chronic active colitis without any granulomas. Simultaneously, colonoscopy showed mild mucosal folds thickening in jejunal and proximal ileal loops with normal appearance of the rest of the bowel loops. The patient was diagnosed with inflammatory bowel disease (IBD) and Crohn's disease (CD). Various treatment modalities were attempted, including fecal microbiota transplantation (FMT), corticosteroids, methotrexate, and infliximab, with limited success and even allergic reactions to some medications. Despite the efforts to manage the CD, the patient's condition deteriorated with weight loss and persistent abdominal pain, leading to hospitalization. Repeated investigations revealed deep ulcers and pseudomembranes in the rectum and sigmoid colon. In light of persistent disease activity, treatments escalated to include ganciclovir, ustekinumab, intravenous steroids, and intravenous immunoglobulin (IVIG). However, the patient continued to experience symptoms and eventually developed limited peritonitis and underwent exploratory laparotomy. During the surgery, extensive involvement of the large bowel and distal ileum was observed. The specimen was sent for histopathological evaluation.

A gross examination of the subtotal colectomy specimen showed diffuse superficial and deep ulcers with pseudopolyp formation involving the cecum, ascending, and transverse colon. The base of the ulcers was filled with necrotic material and covered with slough. A perforation was identified in the cecum measuring 1.5 x 1 cm.

**Figure 2:**
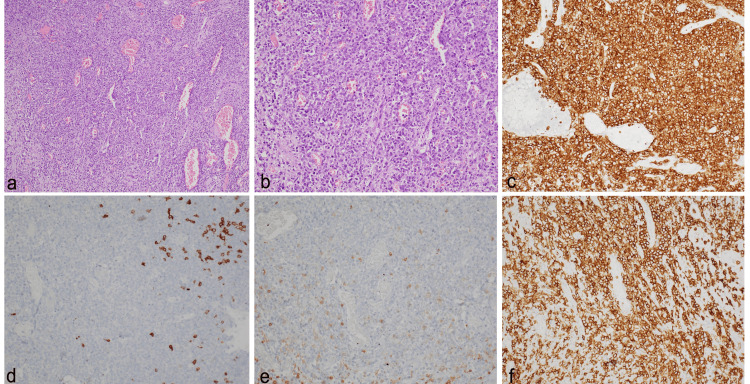
Case Report 2 a: ulcerative lesion infiltrated by medium-sized lymphoid cells (HE, x100); b: lymphoid cells (x400); c: diffuse immunopositivity for CD3 (x200); d: negative for CD20 (x200); e: negative for CD4 (x200); f: positive for CD8 (x200)

Microscopic examination showed transmural infiltration by small- to medium-sized atypical lymphoid cells. There were superficial mucosal ulcerations as well as deep fissuring ulcerations extending up to the muscularis propria. The lesion had a similar angiocentric and angiodestructive pattern inducing extensive coagulative necrosis and mucosal ischemic changes. The atypical cells had coarse granular nuclear chromatin, inconspicuous nucleoli, and scant cytoplasm. In addition, forty-eight lymph nodes dissected from the attached fat were free of disease and showed reactive change only. On immunohistochemistry, the tumor cells are positive for CD3, CD8, and granzyme, whereas negative for CD20 and CD56 (Figure 2). 

Postoperatively, the patient was deemed unfit for chemotherapy due to poor performance status. Throughout the hospitalization, the patient acquired multiple hospital-acquired infections (HAIs), including pneumonia and bloodstream infections caused by Klebsiella pneumoniae, Acinetobacter baumannii, and Stenotrophomonas maltophilia. These infections were managed with appropriate antibiotics, antifungals, and supportive care. Additionally, the patient developed an oral herpetic infection, which was treated with acyclovir. Nutritional support, initially through total parenteral nutrition (TPN), was gradually transitioned to oral intake as the patient's condition improved significantly.

## Discussion

ENKTL develops most commonly in the upper aerodigestive tract. It is mostly seen in Asia and parts of Central and South America. In Asia, it generally affects young males and has a poor prognosis [3]. Intestinal NK/T-cell lymphomas (INKTL) are fairly uncommon, and only a limited number of cases have been reported in the literature. 

Genetic abnormalities and EBV infection play the main role in the etiopathogenesis of this lymphoproliferative disorder. Among the genetic abnormalities, the JAK/STAT pathway is the most important, as it is involved in hematopoiesis and immune development. STAT3 mutations drive phosphorylation and transcriptional activation of STAT3 in the absence of cytokines. Activated STAT3 enhances the expression of PD-L1, which binds to PD-1, an inhibitory receptor on T-cells, leading to suppression of T-cell activation and signaling. This interaction hence promotes tumor cell survival. EBV infection downregulates the expression of NHEJ1, a key DNA repair factor involved in the NHEJ pathway for repairing double-stranded breaks. This disruption causes genome instability, contributing to the development of ENKTL. Through the expression of EBV nuclear antigen 1 (EBNA1), latent membrane proteins (LMP) 1, LMP2A, and LMP2B (from latency phase II), EBV modulates cell signaling and creates barriers to apoptotic signals, helping the virus to evade T-cell-mediated immune responses [4].

INKTL usually presents with abdominal symptoms like chronic diarrhea, pain, and vomiting. Aggressive tumors like enteropathy-associated T-cell lymphoma, monomorphic epitheliotropic intestinal T-cell lymphoma, and extranodal NK/T-cell lymphoma may lead to intestinal obstruction and perforation [1]. Among the few cases reported in the literature, ENKTL presented mostly with chronic diarrhea, often with high fever and emaciation. The endoscopic findings are non-specific, ranging from superficial erosions to ulcerative infiltrating lesions with intervening skip lesions [5]. The adult-onset disease prognosis is poor, and infection and multiple organ failure are the main causes of death. 

There is a significant overlap in the clinical presentation of ENKTL and Crohn’s disease, as both present with abdominal pain and diarrhea. The endoscopic findings are also similar, including multiple ulcerations with skip lesions [3,5]. This often leads the clinician to falsely diagnose an extranodal NK/T-cell lymphoma as Crohn’s disease. On histopathology, the lymphoproliferative proliferation seen in lymphoma may even be misinterpreted as transmural inflammation of Crohn’s disease. Superficial as well as deep ulcers and fibrosis can also be seen in both [6]. Due to a lot of ischemic changes due to the angiodestructive nature of the disease dominating the picture, the atypical cells may be missed. Hence, a very careful examination is required, and subtle hints must be picked up by the pathologist, as well as an extended immunohistochemical panel that should be employed in suspicious cases. 

Most of the patients with primary intestinal NK/T-cell lymphoma in the literature had a poor prognosis, and the average survival period was 2.83-9.5 months [7]. Early onset NK/T-cell lymphomas require radiation therapy only and have a five-year survival of about 70 percent. They are usually localized and are treated effectively. This further emphasizes the need to diagnose this intestinal lymphoma at an earlier stage. Patients with advanced NK/T-cell lymphoma receive radiation therapy synchronously or metachronously with multi-drug chemotherapy, including L-asparaginase, with a five-year survival of up to 40 percent [8]. In recent years, PDL1 immunotherapy in advanced stages of NK/T-cell lymphoma has shown significant improvement and enhancement of survival rate [9]. 

## Conclusions

Primary intestinal NK/T-cell lymphomas are extremely uncommon. There is a significant overlap in the clinical presentation, endoscopic, and imaging findings of Crohn’s disease and intestinal NK/T-cell lymphoma. Multiple endoscopic biopsies with the use of an elaborate immunohistochemical panel should be employed in suspicious cases. Furthermore, in situ hybridization study is very helpful. These can help in diagnosing this entity at an early stage, thereby improving the management and prognosis of the patients. These two cases are being reported to increase the awareness as well as to describe the pitfalls in the diagnosis of extranodal NK/T-cell lymphoma.
